# Effects of Microplastic Accumulation on Neuronal Death After Global Cerebral Ischemia

**DOI:** 10.3390/cells14040241

**Published:** 2025-02-07

**Authors:** Dong Yeon Kim, Min Kyu Park, Hyun Wook Yang, Seo Young Woo, Hyun Ho Jung, Dae-Soon Son, Bo Young Choi, Sang Won Suh

**Affiliations:** 1 Department of Physiology, College of Medicine, Hallym University, Chuncheon 24252, Republic of Korea; roy8596@naver.com (D.Y.K.); bagmingyu50@gmail.com (M.K.P.); akqjqtj5@hallym.ac.kr (H.W.Y.); 1wsy@naver.com (S.Y.W.); wjdgusgh1021@naver.com (H.H.J.); 2 Division of Data Science, Data Science Convergence Research Center, Hallym University, Chuncheon 24252, Republic of Korea; biostat@hallym.ac.kr; 3 Institute of Sport Science, Hallym University, Chuncheon 24252, Republic of Korea; bychoi@hallym.ac.kr; 4 Department of Physical Education, Hallym University, Chuncheon 24252, Republic of Korea

**Keywords:** global cerebral ischemia, microplastic, neuroinflammation, microtubule, myelin sheath, tau protein

## Abstract

Brain ischemia, a condition in which the brain is deprived of blood flow, can lead to a stroke due to blocked or unstable blood vessels. Global cerebral ischemia (GCI), characterized by an interruption in blood flow, deprives the brain of oxygen and nutrients, producing reactive oxygen species (ROS) that trigger cell death, which kills nerve cells. Microplastics (MPs), tiny environmental pollutants, can enter the human body through contaminated food, water, disposable items, cosmetics, and more. Once in the brain, MPs can increase neuroinflammation by overstimulating inflammatory factors such as microglia. MPs can also damage neurons by scratching myelin and microtubules, slowing signal transduction, causing cognitive impairment, and leading to neuronal death. Furthermore, microtubule damage may result in the release of phosphorylated tau proteins, potentially linked to Alzheimer’s disease. We hypothesized that MPs could exacerbate neuroinflammation and microtubule destruction after GCI, leading to increased neuronal death. To test this hypothesis, we administered MPs (0.5 µm) orally at a dose of 50 mg/kg before and after inducing GCI. Staining techniques such as Fluoro-Jade B (FJB), ionized calcium-binding adaptor molecule 1 (Iba-1), cluster of differentiation 68 (CD68), myelin basic protein (MBP), and microtubule-associated protein 2 (MAP2) were used, along with Western blot analysis for interleukin-6 (IL-6), TNF-α, tau-5, and phospho-tau (S396) to evaluate the effects of MPs on neuronal cell death, neuroinflammation, and microtubule destruction. The results showed that MP accumulation significantly increased neuroinflammation, microtubule disruption, and neuronal cell death in the GCI-MP group compared to the GCI-vehicle group. Therefore, this study suggests that MP accumulation in daily life may contribute to the exacerbation of the disease, potentially leading to severe neuronal cell death after GCI.

## 1. Introduction

The human brain is one of the most important organs in the body, and diseases that affect it cause serious problems [1]. Cerebral ischemia, the leading cause of stroke, is caused by blockages or unstable blood flow in the brain’s blood vessels [2]. Ischemia can be categorized into global cerebral ischemia (GCI), which affects entire regions of the brain, and focal cerebral ischemia (FCI), which targets specific areas [3]. In GCI, restricted blood supply to the brain leads to cerebral hypoxia and nutrient deprivation, which causes cell death, neuronal loss, and reactive oxygen species (ROS) production [4,5]. This condition is often caused by atherosclerosis, thrombus blockage, arterial plaque buildup, and especially heart attacks [6,7,8]. Damage to hippocampal neurons caused by GCI is irreversible and manifests as memory impairment, cognitive decline, decreased motor skills, and sensory disturbances [9].

Microtubules are essential intracellular structures involved in maintaining cell morphology and regulating various physiological functions [10]. Composed of tubulin subunits, microtubules dynamically assemble and disassemble to control processes such as intracellular trafficking, cell division, and neural signaling [11]. Ischemia can affect microtubules in several ways; for example, vasoconstriction reduces blood flow, leading to microvascular occlusion due to thrombosis or vessel wall inflammation [12]. Reduced blood supply from microvessels to tissue can result in insufficient oxygen and nutrient supply, which can lead to tissue damage [13]. The release of tau protein from microtubule damage is pathologically altered and is associated with both ischemia itself and dysfunction of the ischemic blood–brain barrier [14]. Tau proteins undergo phosphorylation at numerous amino acid residues, particularly at serine (Ser), threonine (Thr), and tyrosine (Tyr) residues in their side chains [15]. This phosphorylation, catalyzed by ATP-using protein kinases, regulates the stability and physical properties of microtubules to maintain cellular structure and modulate intracellular signaling pathways by regulating interactions between various signaling molecules [16]. Dysregulation or excessive phosphorylation of tau protein is associated with neurodegenerative diseases, most notably in Alzheimer’s disease, where abnormal phosphorylation of tau protein is observed to correlate with microtubule abnormalities and cell death [17,18].

Microplastics have become a fast-growing environmental problem that has attracted the attention of scientists, policymakers, and the general public [19]. These tiny particles, measuring less than 5 mm in size, are found in a variety of ecosystems, from the depths of the ocean to the tops of mountains [20]. Whether they originate from the degradation of larger plastic products or from microbeads in personal care products, microplastics pose a serious threat to marine life and human health [21]. Once in the environment, microplastics penetrate ecosystems, including the ocean depths, soil, and plants, and travel up the food chain [22]. Their small size and buoyancy make them easily ingested by a wide range of marine organisms, from zooplankton to top-level predators, promoting the bioaccumulation of toxins and other harmful substances [23]. Despite their widespread prevalence, the full extent of the risks associated with microplastics remains poorly understood. Recent studies have shown that microplastics can disrupt physiological processes and reproductive functions in marine organisms [24], as well as negatively impact overall marine ecosystems [25]. Furthermore, the potential for microplastics to damage microtubules and contribute to disease poses a significant public health concern. The mechanisms through which microplastics destabilize microtubules include physical abrasion and interference with tubulin polymerization, which contribute to a variety of diseases, particularly neurodegenerative disorders. More research is needed to fully characterize the pathways through which microplastics exert their toxic effects and to develop interventions to protect human health. This includes understanding how microplastics interact with cellular structures, identifying biomarkers for early detection of microplastic-related damage, and developing strategies to mitigate exposure and impacts. Public awareness and policy action are also essential to reduce plastic pollution, and their health effects as vectors of pathogens and persistent organic pollutants emphasize their importance as an environmental pollutant of concern [26]. From a medical and physiological standpoint, the effects of microplastics on human health are particularly concerning. Microplastics can enter the human body through the consumption of contaminated food and beverages, leading to their accumulation in the digestive tract [27,28,29,30]. Furthermore, when microplastics carrying toxic substances on their surfaces are ingested, they may disrupt metabolic processes, alter hormone levels, and impair immune function, thereby posing significant risks to overall human physiology [31]. Recent studies have raised concerns about the effects of microplastics on the brain, including that microplastic particles can pass through the bloodstream and potentially damage brain cells [32]. Microplastics can also directly affect brain tissue by blocking brain blood vessels or adsorbing toxins, causing nerve cell damage [33]. These effects can exacerbate cognitive decline and increase the risk of neurodegenerative diseases [34].

Microplastics can also damage nerve myelin sheaths within brain tissue through mechanical interference, which can disrupt the neural skeleton [35]. These disruptions impair the transmission of electrical signals and compromise the structural integrity of nerve cells [36]. As a result, microplastics can adversely affect the health and function of nerve cells, exacerbating cognitive impairment and increasing the risk of neurodegenerative diseases [37]. In conclusion, this study aims to explore the multiple impacts of microplastics on marine ecosystems and human health. By elucidating the mechanisms of microplastic toxicity and assessing their ecological and physiological impacts, we hope to propose evidence-based policies and interventions to mitigate the harmful effects of microplastic pollution. Such a comprehensive understanding is crucial to developing effective strategies for protecting both the environment and public health from the pervasive threat of microplastics.

## 2. Materials and Methods

### 2.1. Experimental Animals

Animal care and handling followed the guidelines established by the National Institutes of Health. All experimental procedures were approved by the Institutional Animal Care and Use Committee of Hallym University College of Medicine (protocol number Hallym 2022-69). Male Sprague Dawley (SD) rats (310–320 g body weight, 8 weeks old; Daehan Bio Link (DBL), Eumseong-gun, Chungcheongbuk-do, Republic of Korea) were used in this study. The animals were housed in a controlled animal care facility with constant temperature (22 ± 2 °C), humidity (55 ± 5%), and a 12 h light/dark cycle. For histological analysis, brain tissue staining was performed using the following groups: Sham-Vehicle (*n* = 5), Sham-Microplastic (*n* = 5), GCI-Vehicle (*n* = 8), and GCI-Microplastic (*n* = 9). For protein quantification via Western blot analysis, the experiment included the following groups: Sham-Vehicle (*n* = 5), Sham-Microplastic (*n* = 5), GCI-Vehicle (*n* = 5), and GCI-Microplastic (*n* = 5). For behavioral and cognitive function assessments, including the modified neurological severity score (mNSS) and Morris water maze (MWM) tests, the following groups were used: Sham-Vehicle (*n* = 5), Sham-Microplastic (*n* = 5), GCI-Vehicle (*n* = 5), and GCI-Microplastic (*n* = 5).

### 2.2. Experimental Schedule

The group designated for histological analysis received oral administration of microplastics at a concentration of 50 mg/kg for one week. Following the induction of global cerebral ischemia (GCI), microplastic administration continued for an additional week, totaling two weeks of exposure. After this period, the animals were sacrificed, and brain tissue was collected. The group used for protein quantification via Western blot analysis received oral administration of microplastics at 50 mg/kg for one week. Three days after GCI induction, the animals were sacrificed, and hippocampal tissue was collected. The group undergoing behavioral and cognitive function tests received oral administration of microplastics at 50 mg/kg for one week, followed by an additional week of administration after GCI induction, totaling two weeks of exposure. In the third week, behavioral function was assessed using the modified neurological severity score (mNSS) and Morris water maze (MWM) tests. In the fourth week, cognitive function was further evaluated using the MWM test.

### 2.3. Global Cerebral Ischemia Disease Modeling

To investigate whether microplastics exacerbate brain injury and attenuate the neuroprotective effects induced by global cerebral ischemia (GCI), we used 8-week-old SD rats. Rats were anesthetized with 2–3% isoflurane and ventilated with a mixture of nitrous oxide (70%) and oxygen (30%). Body temperature was continuously monitored throughout the procedure using a temperature-monitoring heating pad (Harvard Apparatus, Holliston, MA, USA) to prevent hypothermia. A polyethylene tube was inserted into the right femoral artery for arterial blood pressure monitoring and temporary blood drainage during hypotension. Using a surgical microscope (SZ61, Olympus, Shinjuku, Japan), the bilateral common carotid arteries (BCCAs) were isolated from the vagus nerve. Electrodes were then inserted into the bilateral burr holes to continuously monitor the electroencephalogram (EEG). Approximately 7 min after bilateral CCA clamping and femoral artery blood drainage (maintaining blood pressure within the range of 50 mmHg systolic and 40 mmHg diastolic), isoelectric activity appeared on the EEG. The CCAs were then unclamped and blood reperfusion was initiated to restore cerebral circulation. Sham surgery was performed by performing skin incision and cannulation of the femoral artery and bilateral common carotid arteries without occluding the femoral artery.

### 2.4. Microplastic Administration

Normal microplastics (polystyrene, 0.5 µm; product: PST500, Lab261, 265 Cambridge Avenue #60601, Palo Alto, CA, USA) and fluorescently labeled microplastics (polystyrene, 0.5 µm; product: G500, Thermo Scientific, Waltham, MA, USA) were administered orally at a dose of 50 mg/kg using a sonde for 1 week before and after GCI induction. The microplastics were suspended in sterile saline, while rats were provided food and water ad libitum.

The 0.5 µm size was selected because microplastics smaller than 2 µm can cross the blood–brain barrier (BBB). The microplastics were supplied as a 1% solid suspension (10 mg/mL), with a small amount of surfactant in deionized water and 2 mM sodium azide as an antimicrobial agent.

### 2.5. Brain Tissue Sample Preparation

Brain tissue sampling for analysis was conducted as follows: Rats subjected to ischemia were anesthetized with urethane (1.5 g/kg, i.p.) and perfused through the heart first with saline to remove blood, followed by 4% paraformaldehyde (PFA) to fix the tissues. After perfusion, the brains were quickly harvested and further fixed in 4% PFA for 1 h to enhance tissue preservation. The PFA was then replaced with a 30% sucrose solution, and the brains were stored in the solution until they sank to the bottom, ensuring optimal tissue protection for cryosectioning. The brains were subsequently frozen and sectioned into 30 µm thick slices using a cryostat microtome (CM1850, Leica, Wetzlar, Germany). To evaluate the effects of microplastic ingestion and ischemia on memory, learning, and cognitive function, targeted regions such as CA1, CA2, and the subiculum were analyzed. Brain samples were collected starting 3.0 mm posterior to the bregma, with coronal sections obtained at 0.25 mm intervals. A total of 7–8 sections were collected, and 6 representative coronal sections were selected for analysis. The sections were stored in 2 mL tubes containing appropriate preservation solutions to maintain tissue integrity and prevent degradation. This procedure ensured the proper fixation, preservation, and preparation of brain tissue from ischemic rats for subsequent analysis.

### 2.6. Processing for Immunofluorescence

To perform immunostaining, sectioned brain tissues were washed three times with 0.01 M phosphate-buffered saline (PBS) for 10 min each to remove impurities. Residual blood was removed by pretreating the tissues with 1.2% hydrogen peroxide for 15 min, followed by additional washing with 0.01M PBS. The tissues were then incubated overnight at 4 °C with primary antibodies diluted in PBS containing 0.3% Triton X-100. The primary antibodies used in this study included Mouse anti-SMI-71 (1:500; COVANCE #SMI-71R), Goat anti-Iba-1 (1:500; Abcam, Cambridge, UK, #ab50876), Mouse anti-CD68 (1:100; Abcam, Cambridge, UK, #ab53444), MAP2 (1:400; Certificate of Analysis, #MAB3418), and Rabbit anti-MBP (1:500; Invitrogen, Grand Island, NY, USA, #AB_2736178). After overnight incubation, the tissues were thoroughly washed with 0.01 M PBS to remove unbound primary antibodies. The tissues were then incubated at room temperature for 2 h with secondary antibodies, including Alexa-Fluor-594-conjugated IgG and Alexa-Fluor-488-conjugated IgG, both diluted 1:250 (Invitrogen, Grand Island, NY, USA). To visualize cell nuclei, the tissues were stained with DAPI (4′,6-diamidino-2-phenylindole), diluted 1:1000 in PBS. The stained tissues were mounted on gelatin-coated slides and secured with DPX mounting medium (Sigma-Aldrich). Fluorescence imaging was performed using a fluorescence microscope (Olympus BX53T-32X01/FL with DP74-ST, Tokyo, Japan) and a confocal microscope (Carl Zeiss LSM710, Oberkochen, Germany). The fluorescence intensity of the stained sections was quantified and analyzed using ImageJ software v.1.47c (NIH, Bethesda, MD, USA).

### 2.7. Immunohistochemistry for Diaminobenzidine (DAB) Methods

To perform immunohistochemical analysis, pre-treatment steps were conducted to optimize antibody–antigen interactions. Brain sections were incubated with the primary antibody, NeuN (1:500 dilution, Millipore Co., Burlington, MA, USA), in PBS containing 0.3% Triton X-100 at room temperature for 2 h. After primary antibody incubation, the tissues were thoroughly washed with PBS and incubated with the secondary antibody, anti-rabbit IgG (1:250 dilution, Jackson Immunoresearch Inc., West Grove, PA, USA), under the same conditions to ensure specific binding. The sections were subsequently washed and incubated in ABC solution (Vector Laboratories, Burlingame, CA, USA) for 2 h to amplify the immunoreactive signal. Following ABC treatment, the sections were incubated for 1 min and 30 s in a solution containing 0.01 M PBS, 30% H_2_O_2_ (50 µL per 100 mL), and 0.06% 3,3′-diaminobenzidine (DAB) (Sigma-Aldrich Co., St. Louis, MO, USA). This reaction produced a brown precipitate, enabling the visualization of antigen–antibody complexes. After staining, the brain sections were mounted on gelatin-coated slides and examined under a light microscope to confirm staining patterns. Quantitative analysis of staining intensity was conducted using ImageJ software v.1.47c (NIH, Bethesda, MD, USA). Quantitative assessment of IgG leakage in the hippocampal regions (subiculum, CA1, and CA2) was performed by a blinded observer using ImageJ. The analysis involved the following steps: Image Conversion: Images were loaded into ImageJ and converted to grayscale (Image → Type → 8-bit). Grayscale was inverted (Edit → Invert) to enhance visualization. Region of Interest (ROI) Measurement: Selected ROIs representing IgG leakage were measured (Analyze → Measure), and the results were recorded. Mean Gray Value: The mean gray value was calculated as a quantitative measure of IgG leakage. This approach ensured reproducibility and accuracy in the evaluation of immunohistochemical data.

### 2.8. Confirmation of Neuron Death

To evaluate neuronal damage following ischemia, 30 µm thick brain sections were mounted on gelatin-coated slides (Fisher Scientific, Pittsburgh, PA, USA) for staining. Degenerating neurons were visualized using the Fluoro-Jade B (FJB) staining protocol as previously described. The staining process involved sequential immersion of the slides in 100% ethanol for 3 min, 70% ethanol for 1 min, and distilled water for 1 min, followed by treatment with 0.06% potassium permanganate solution for 15 min. The slides were then incubated in 0.001% FJB solution (Histo-Chem Inc., Jefferson, AR, USA) for 30 min and washed three times with distilled water for 10 min each. After washing, the slides were gently air-dried using a controlled air stream (Labtech Co., Ltd., Namyangju, Republic of Korea), dehydrated in xylene for 2 min, and cover-slipped with DPX (Sigma-Aldrich Co., St. Louis, MO, USA). Fluorescent staining was examined under a fluorescence microscope (Olympus, Japan) using blue light excitation (450–490 nm). Approximately eight coronal brain sections were selected per animal for analysis. FJB-positive cells were quantified by a blinded observer in the hippocampal regions, including the subiculum, CA1, and CA2 areas of both hemispheres. The total number of FJB-positive cells in the hippocampal region was calculated and subjected to statistical analysis. Special attention was given to ensure uniform staining and consistent cell distribution to maintain the reliability of neuronal degeneration quantification [38]. To address the potential issue of autofluorescence in paraformaldehyde-fixed tissue, we performed additional control experiments. Specifically, we included negative controls without Fluoro-Jade B staining and imaged these samples to assess background fluorescence in the green channel. This allowed us to differentiate autofluorescence from specific staining.

### 2.9. Western Blot Analysis

Rat brain hippocampus samples were lysed in lysis buffer containing protease inhibitor (11697498001, Sigma, Burbank, CA, USA), phosphatase inhibitor (4906845001, Sigma, USA), and RIPA buffer (IBS-BR002, iNtRON Seongnam, Republic of Korea). The samples were then centrifuged at 14,000 rpm for 20 min at 4 °C. Supernatant proteins were quantified using the Bradford protein assay. The quantified supernatant was boiled in SDS loading buffer and then separated by SDS-PAGE, and the separated proteins were transferred to a PVDF membrane. Primary antibodies against the following target antigens were used: IL-6 (Abcam, 1:1000, #ab9324), Tau-5 (Abcam, 1:1000, #ab80579), P-Tau (Abcam, 1:1000, #ab109390), TNF-α (Abcam, 1:500, #ab6671), and β-Actin (1:10,000, #3700, Cell Signaling Technologies, Danvers, MA, USA).

### 2.10. Behavior Testing

#### 2.10.1. Modified Neurological Severity Score (mNSS)

To evaluate the neurological function and potential toxicity of microplastics following the induction of global cerebral ischemia (GCI) in rats, we administered the modified neurological severity score (mNSS) test. The mNSS is a comprehensive tool used to assess functional impairments resulting from neurological injury, encompassing sensorimotor deficits, motor function, sensory function, balance, and reflex responses. Scores range from 0 to 18, where 0 denotes no neurological impairment and 18 signifies the most severe level of neurological dysfunction. The mNSS assessment was initiated 4 h post-GCI induction and continued daily for a duration of 7 days. Each rat was evaluated using the mNSS criteria, with 1 point assigned for each identified neurological deficit. These scores were systematically recorded and analyzed to monitor the progression of neurological impairment over the evaluation period.

#### 2.10.2. Morris Water Maze (MWM)

To evaluate the impact of microplastic ingestion on brain damage induced by global cerebral ischemia (GCI), we employed the Morris water maze (MWM) test over a duration of 5 days. The MWM test is a well-established behavioral assay used to assess spatial learning and memory in rodents [39]. This assessment was initiated one week post-GCI induction. The experiment was conducted in a circular water-filled tank divided into four quadrants, with a submerged platform located in the center of quadrant 1. Each rat underwent four trials per day, with each trial allowing a maximum of 120 s for the rat to locate the hidden platform. We recorded the escape latency (time taken to find the platform) and the distance traveled by the rats during each trial. Data collection focused on metrics such as escape latency and path length to the target. These metrics were subsequently analyzed using SMART video tracking software 3.0 (Panlab, Carrer de l’Energia, Spain) to assess spatial learning and memory performance in the context of microplastic exposure following GCI.

### 2.11. Quantification of Data

First, brain slices were analyzed using Adobe Photoshop CS5 software v.25.0 (Adobe System Incorporated, San Jose, CA, USA) to quantify the number of immunoreactive and dye-stained tissues. Images were processed using the Analysis/Count tool to identify positive and reactive cells. Second, to quantify the intensity of immunofluorescence, we evaluated the hippocampal region within the brain slices using ImageJ software v.1.47c (National Institutes of Health, Bethesda, MD, USA). Images were converted to 8-bit grayscales using the Image/Color/Channel Split function, and the average gray value, which represents the intensity of immunofluorescence, was calculated using the Analyze/Measure function

### 2.12. Data Analysis

Statistical analyses were performed using nonparametric methods and blind tests to assess differences between the experimental groups. All data are presented as mean ± standard error of the mean (SEM). A *p*-value of less than 0.05 was considered statistically significant. Comparisons between the global cerebral ischemia vehicle group and the microplastic group were conducted using the Mann–Whitney U test. For analyses involving the four experimental groups, statistical significance was assessed using the Kruskal–Wallis test followed by Bonferroni correction.

## 3. Results

### 3.1. Administration of Microplastics Induced Neuronal Death by Accumulating in Microglial and the Blood–Brain Barrier

**Figure 1:**
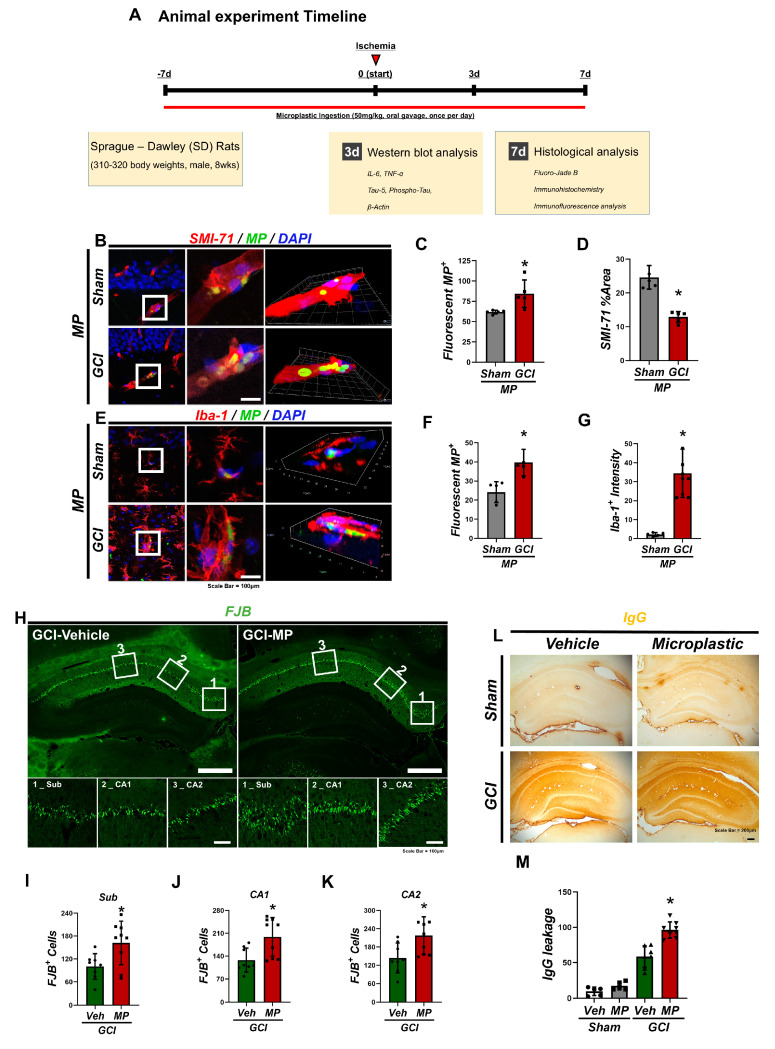
Administration of microplastics induced neuronal death by accumulating in microglial and the blood–brain barrier. (**A**) Experimental timeline for one-week-long animal experiments. (**B**) Representative micrograph illustrating the accumulation of fluorescently labeled microplastics in cerebral vasculature. (**C**) Bar graph comparing the count of microplastics between the GCI group (received fluorescent microplastics) and the Sham group. (**D**) Survival rates of cerebral vessels showing significantly lower vascular survival rates in the MP ingestion group compared to the Sham-administered group, alongside significantly higher MP accumulation (* *p* < 0.05). Data are presented as mean ± SEM and analyzed using the Kruskal–Wallis test (chi-square value = 54.715, degrees of freedom = 3, *p* = 0.001). Scale bar: 100 µm. (**E**) Representative micrograph illustrating the activation rate of microglia and fluorescently labeled MP accumulation. (**F**) Bar graph indicating the number of microplastics between the MP-administered GCI group and the Sham group. (**G**) Microglial activation rate in the CA1 region, with significantly higher microglial activation rate in the GCI group compared to the Sham group, along with significantly higher MP accumulation in the MP ingestion group (* *p* < 0.05). Data are presented as mean ± SEM and analyzed using the Kruskal–Wallis test (chi-square value = 54.715, degrees of freedom = 3, *p* = 0.001). Scale bar: 100 µm. (**H**) Representative micrograph illustrating neuron survival through scratch assay and MP ingestion. (**I**) Neuron survival rates in the subiculum. (**J**) Neuron survival rates in the CA1 region. (**K**) Neuron survival rates in the CA2 region. These graphs demonstrate significantly lower neuron survival rates in the MP ingestion GCI group compared to the GCI-Veh group (* *p* < 0.05). Data are presented as mean ± SEM and analyzed using the Kruskal–Wallis test (chi-square value = 56.400, degrees of freedom = 3, *p* = 0.001). Scale bar: 100 µm. (**L**) Representative micrograph demonstrating the extent of blood–brain barrier (BBB) disruption caused by ischemic injury and microplastic (MP) accumulation. (**M**) Bar graph showing the extent of IgG leakage across the entire hippocampal region. In the Sham-operated group, no IgG leakage was observed, indicating an intact BBB. However, in the ischemia-induced GCI group, severe IgG leakage was detected due to BBB disruption. Importantly, the group with MP ingestion exhibited significantly higher levels of IgG leakage compared to the non-MP ingestion group (* *p* < 0.05), suggesting that microplastic accumulation, when combined with ischemia, worsens BBB damage. The data are presented as mean ± SEM and analyzed using the Kruskal–Wallis test (chi-square value = 60.238, degrees of freedom = 3, *p* = 0.001). Scale bar: 200 µm.

Microplastics (MPs) have been shown to penetrate the blood–brain barrier (BBB) and accumulate in brain blood vessels and microglia, contributing to neurodegeneration. In this study, we investigated the effects of MPs Mon neuronal damage in the hippocampus following global cerebral ischemia (GCI). Experimental rats were orally administered MPs (2 µm, 50 mg/kg) before and after GCI induction. Rats were sacrificed 7 days post-GCI, and both hemispheres were examined histologically (Figure 1A). Fluorescently labeled MPs and the endothelial BBB marker SMI-71 were used to evaluate MP penetration into the brain and endothelial BBB damage in hippocampal regions of interest, including the subiculum (Sub), cornu ammonis 1 (CA1), and CA2. MPs were detected in the CA1 region of MP-treated rats, with greater MP penetration observed in the GCI group (Figure 1B). The MP-administered group also exhibited significantly increased endothelial BBB damage compared to the vehicle-administered group (Figure 1C,D). Phagocytosis of MPs by microglia and other glial cells was confirmed using Iba-1 staining (Figure 1E–G). Neuronal damage caused by MP infiltration was assessed through Fluoro-Jade B (FJB) staining, which revealed a significant increase in degenerative hippocampal neurons in the MP-treated group compared to the vehicle-treated group after cerebral ischemia (Figure 1H–K). Further, fluorescently labeled MP was used to confirm its direct infiltration into the hippocampal region through the BBB. MPs were localized within microglia and adjacent blood vessels, suggesting that MPs promote neuronal destruction and exacerbate neuronal damage (Figure 1L,M). These findings indicate that MP administration contributes to neurodegeneration by infiltrating the brain, damaging the BBB, and interacting with glial cells. This study highlights the neurodegenerative effects of MPs and their role in exacerbating brain damage, emphasizing the need for further research to understand and mitigate MP accumulation in the body.

### 3.2. Microplastic Accumulation Increased Pro-Inflammatory Factors, Particularly in Microglia, After GCI

**Figure 2:**
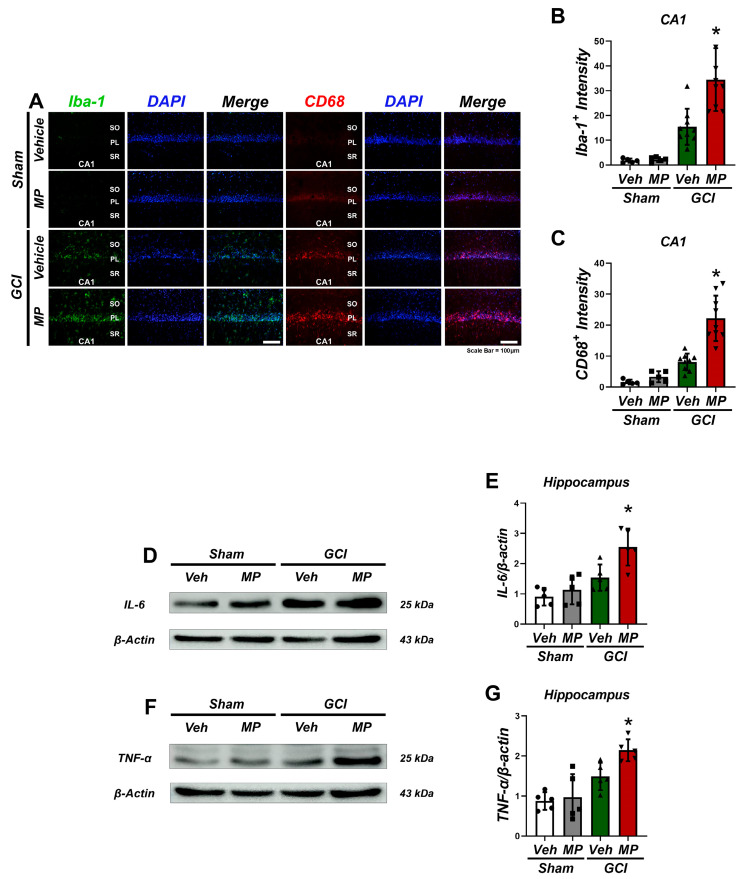
Microplastics increased microglia activation following GCI. (**A**) Immunofluorescence staining for Iba-1 and CD68 was conducted to investigate the heightened activation of microglia following global cerebral ischemia, particularly in response to microplastic ingestion. (**B**,**C**) The bar graphs illustrate a comparison of CD68 and Iba-1 levels between the GCI-MP and GCI-Veh groups in the hippocampal CA1 region, highlighting statistically significant differences (* *p* < 0.05 Iba-1 data, presented as mean ± SEM, were meticulously analyzed using the Kruskal–Wallis test, revealing significant variations (CA1: chi-squared value = 20.408, df = 3, *p* = 0.001). Similarly, CD68 data, also depicted as mean ± SEM, underwent rigorous analysis via the Kruskal–Wallis test, unraveling significant distinctions (CA1: chi-squared value = 22.69, df = 3, *p* = 0.001). The scale bar (100 µm) provides context for the microscopic observations, ensuring precise interpretation. Additionally, sample sizes, comprising *n* = 5 for each sham group and *n* = 8 for each GCI group, reinforce the robustness of the findings. (**D**,**F**) Western blot analysis targeting the inflammation cytokines elucidates the inflammatory response post microplastic ingestion and subsequent ischemic stroke. Labels indicate the molecular weight of each band on the blots and gel images. (**E**) The bar graphs offer a comprehensive overview of the protein levels of IL-6 within the hippocampus, revealing nuanced variations. (**G**) Similarly, the distinct bar graph dedicated to TNF-α protein levels sheds light on its role in the observed inflammatory cascade. Each dataset, meticulously analyzed using the Kruskal–Wallis test (chi-squared value = 8.862, df = 3, *p* = 0.031), underscores the statistical significance denoted by * *p* < 0.05. This rigorous analytical approach, coupled with the precise presentation of data as mean ± SEM, underscores the reliability and robustness of the findings, thereby enhancing the credibility of the study.

To evaluate changes in inflammation after global cerebral ischemia (GCI) induced by microplastic (MP) administration, brain sections were stained with ionized calcium-binding adaptor molecule 1 (Iba-1) and cluster of differentiation 68 (CD68) 7 days after GCI induction to observe inflammation before and after (Figure 2A). The MP-administered group showed increased numbers and activation levels of microglia in the hippocampal subiculum (Sub), cornu ammonis 1 (CA1), and CA2 regions compared to the sham- and vehicle-administered GCI groups (Figure 2B,C). Co-staining showed that microglia were positive for both Iba-1 and CD68. Although activation was observed in the GCI group without MP administration, the signal was less compared to the MP-administered group (Figure 2B,C). Furthermore, Western blot analysis showed that the MP-administered group had increased lymphocyte production and elevated levels of the cytokines IL-6 and TNF-α compared to the sham group (Figure 2D–G). These results suggest that MP administration may exacerbate the neuroinflammatory response associated with cerebral ischemia through microglia activation and increased cytokine production. This highlights the potential for understanding the mechanisms of brain injury and exploring new therapeutic approaches to neuroinflammation.

### 3.3. Microplastics Cause Dendritic Spine and Synaptic Protein Loss by Damaging Myelin Sheaths and Microtubule-Associated Proteins, Impacting the Axonal Skeleton

**Figure 3:**
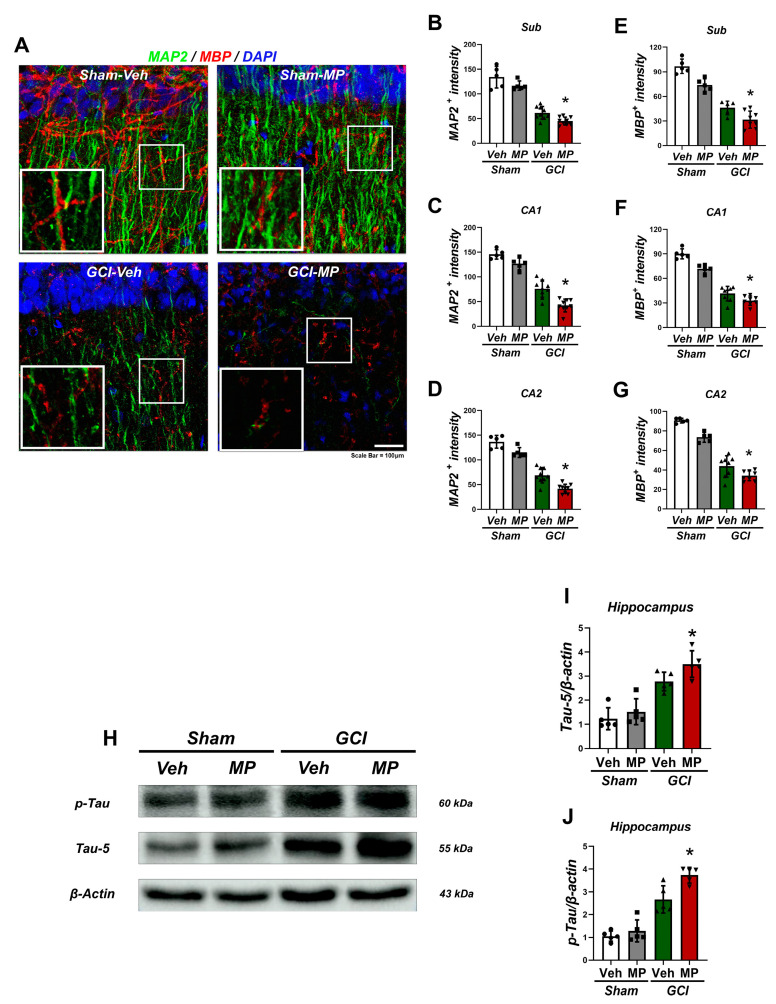
Microplastics increased microtubule and myelin sheath damage after GCI. (**A**) The representative images illustrate hippocampal microtubule damage (MAP2) and myelin sheath damage (MBP) in the CA1 region seven days after global cerebral ischemia (GCI). The MP-administered GCI group exhibits exacerbation of MAP2 and MBP signals compared to the vehicle-administered GCI group. Scale bar = 100 µm. (**B**–**G**) Quantitative analysis confirmed that MAP2 and MBP fluorescence intensity was reduced in the MP-administered GCI group compared to the other groups. Data are reported as mean ± SEM, and statistical analysis was conducted using the Kruskal–Wallis test followed by Bonferroni post hoc test. The chi-square values and degrees of freedom are as follows: (**B**): chi-square = 22.063, df = 3, *p* < 0.0001; (**C**): chi-square = 23.086, df = 3, *p* < 0.0001; (**D**): chi-square = 23.086, df = 3, *p* < 0.0001; (**F**): chi-square = 20.007, df = 3, *p* < 0.0001; (**G**): chi-square = 22.583, df = 3, *p* < 0.0001; Statistical significance is denoted by * *p* < 0.05. The study included a sample size of *n* = 5 for all sham-operated groups, *n* = 8 for the vehicle-administered GCI group, and *n* = 8 for the MP-administered GCI group. (**H**) Western blot analysis for Tau and phospho-Tau quantifies the increase in microtubule damage and tau phosphorylation signaling following MP ingestion pre- and post-stroke. Labels indicate the molecular weight of each band on the blots and gel images. (**I**,**J**) Bar graphs depict the levels of Tau and phospho-Tau proteins in the hippocampus. Each sample size is *n* = 5 for both sham and GCI groups. Statistical significance is indicated by * *p* < 0.05, and data are analyzed using the Kruskal–Wallis test, reporting mean ± SEM (chi-square value = 8.862, degrees of freedom = 3, *p* = 0.031).

Induction of global cerebral ischemia (GCI) for 7 days resulted in pronounced demyelination and microtubule disruption with loss of synaptic proteins. This was assessed in brain sections stained for myelin basic protein (MBP) and microtubule-associated protein 2 (MAP2) (Figure 3A). Immunohistochemistry revealed a significant increase in microtubule damage in the MP-administered sham group and GCI-administered group compared to the sham group. Quantitative analysis showed decreased positive area staining for MAP2 in the subiculum (Sub), cornu ammonis 1 (CA1), and CA2 regions (Figure 3B–D). Myelin sheath damage, assessed by MBP staining, also showed increased damage in the GCI-Veh group compared to the Sham-Veh group in the hippocampal Sub, CA1, and CA2 regions (Figure 3E–G). Notably, the MP-administered group showed more extensive sheath loss compared to the vehicle and sham groups. Quantitative analysis showed decreased positive areas of myelin cis protein in the Sub, CA1, and CA2 regions. MP administration resulted in additional phosphorylation of tau protein compared to the GCI-Veh group, as evidenced by an increase in phosphorylated tau protein (Figure 3H–J). These results suggest that MP exposure exacerbates the neuropathological effects of GCI, leading to demyelination, microtubule disruption, and increased phosphorylation of the tau protein. This indicates a potentially more severe neurodegenerative response and highlights the need for further research to mitigate the harmful effects of microplastics on brain health.

### 3.4. Microplastics Aggravate Motor and Cognitive Impairment After Global Cerebral Ischemia

**Figure 4:**
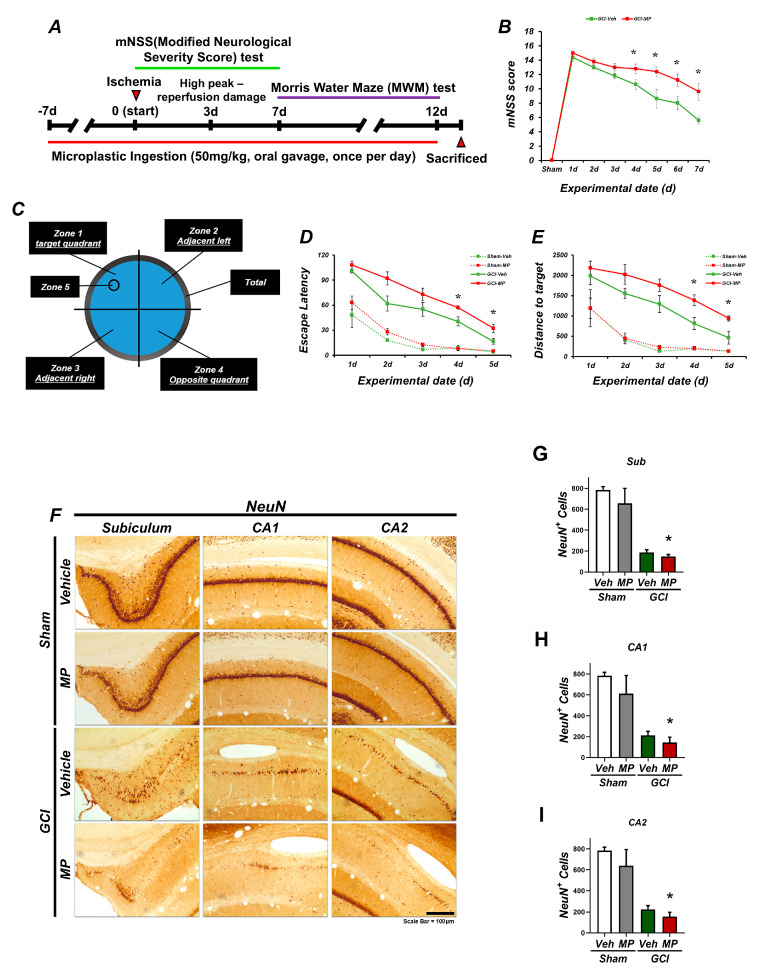
Microplastics caused motor and cognitive impairment after GCI. (**A**) The experimental timeline involved administering MPs for a total duration of 12 days, starting 7 days prior to either GCI or sham surgery and continuing until 13 days post-surgery. (**B**) The modified neurological severity score (mNSS) assessment was carried out at various post-GCI time points, with scores ranging from 0 to 18. Significant differences between GCI-MP and GCI-Veh were identified as early as 4 days after mNSS testing began. (**C**) Schematic representation of the Morris water maze (MWM) tank. (**D**) Throughout the 5-day acquisition trial period, records were kept for escape latency and distance to the target, revealing a significant increase in the GCI-MP group compared to the GCI-Veh group. (**E**) Graph illustrating the cumulative swimming distance to the target over 5 consecutive days. Data are presented as mean ± SEM (repeated measures of ANOVA: mNSS test-GCI group, time: F = 52.395, *p* < 0.0001; group: F = 205.808, *p* < 0.0001; time × group: F = 2.169, *p* = 0.043. MWM test-escape latency: GCI group, time: F = 10.344, *p* < 0.0001; group: F = 73.820, *p* < 0.0001; time × group: F = 3.203, *p* = 0.019. Distance to target: GCI group, time: F = 19.687, *p* < 0.0001; group: F = 109.387, *p* < 0.0001; time × group: F = 4.069, *p* = 0.006. (**F**) Representative NeuN immunostaining image for assessing hippocampal neuronal survival post-GCI. Scale bar = 100 µm. (**G**–**I**) Bar graphs quantifying NeuN-positive neurons in the hippocampal subregions (subiculum, CA1, and CA2). Data are presented as mean ± SEM (Kruskal–Wallis test, followed by Bonferroni post-hoc test: H: chi-square = 16.820, df = 3, *p* = 0.001; I: chi-square = 22.076, df = 3, *p* < 0.0001). Statistical significance is denoted by * *p* < 0.05. Sample size: *n* = 5 for all sham surgery groups and *n* = 8 for all GCI groups.

To evaluate the effects of GCI induction and MP administration on behavior, we performed modified neurological severity score (mNSS) and Morris water maze (MWM) tests. mNSS tests were performed from day 0 to day 7 after GCI induction or sham surgery (Figure 4A). MWM tests were then performed from day 7 to day 12, and all rats were sacrificed on day 13. Neurological deficits were assessed using mNSS, with a score of ‘10’ indicating severe neurological impairment. Comparing the MP-administered group to the vehicle (Veh) group after GCI induction, MP administration increased the mNSS score, indicating neurological deficits. The mNSS test assessed motor function, sensory reflexes, and balance, showing that GCI induction exacerbated neurological deficits due to MP administration (Figure 4B). The MWM test was used to assess cognitive and memory function (Figure 4C). The GCI group receiving MP had significantly increased escape latency and total swimming distance to the target compared to the GCI-Veh group (Figure 4D,E). These results suggest that MP ingestion with GCI exacerbates cognitive and memory impairment. To further investigate the long-term neurotoxic effects of MP administration, we stained for the neuronal marker NeuN after 2 weeks of behavioral testing (Figure 4F). A decrease in NeuN-positive cells was observed in the hippocampal cerebellum (Sub), cornu ammonis 1 (CA1), and CA2 regions of the hippocampus in the GCI-Veh group compared to the sham-Veh group. However, the GCI-MP group showed a more pronounced reduction in NeuN-positive cells in hippocampal regions. Quantitative analysis confirmed that the number of NeuN-positive cells was reduced in the hippocampal sub, CA1, and CA2 regions after MP administration. Overall, these findings indicate that GCI induction with MP administration exacerbates behavioral deficits, including motor dysfunction and cognitive impairment (Figure 4G–I). Furthermore, prolonged MP administration results in neuronal damage, as evidenced by a decrease in the number of NeuN-positive cells in hippocampal regions. These results emphasize the detrimental effects of microplastics (MPs) on neural function and highlight the need for further investigation into mechanisms of action and neuroprotective strategies. These investigations will play a pivotal role in developing a deeper understanding of the neurological effects of microplastics and the overall understanding of brain health under environmental pollution. Furthermore, research is needed on proactive and interventional measures to mitigate the potential risks posed by MPs.

## 4. Discussion

Micro and nano plastics are becoming increasingly prevalent in a variety of environments, including oceans, rivers, soil, and even the atmosphere. Their ubiquitous presence causes numerous environmental problems, including physical harm from ingestion by marine and terrestrial organisms, chemical exposure, and trophic transfer through the food chain [40,41]. In humans, these particles have been detected in drinking water, food, and even the air we breathe, raising concerns about potential health effects, including respiratory and gastrointestinal problems, and widespread systemic effects from chemical contaminants associated with plastics [42,43].

**Figure 5:**
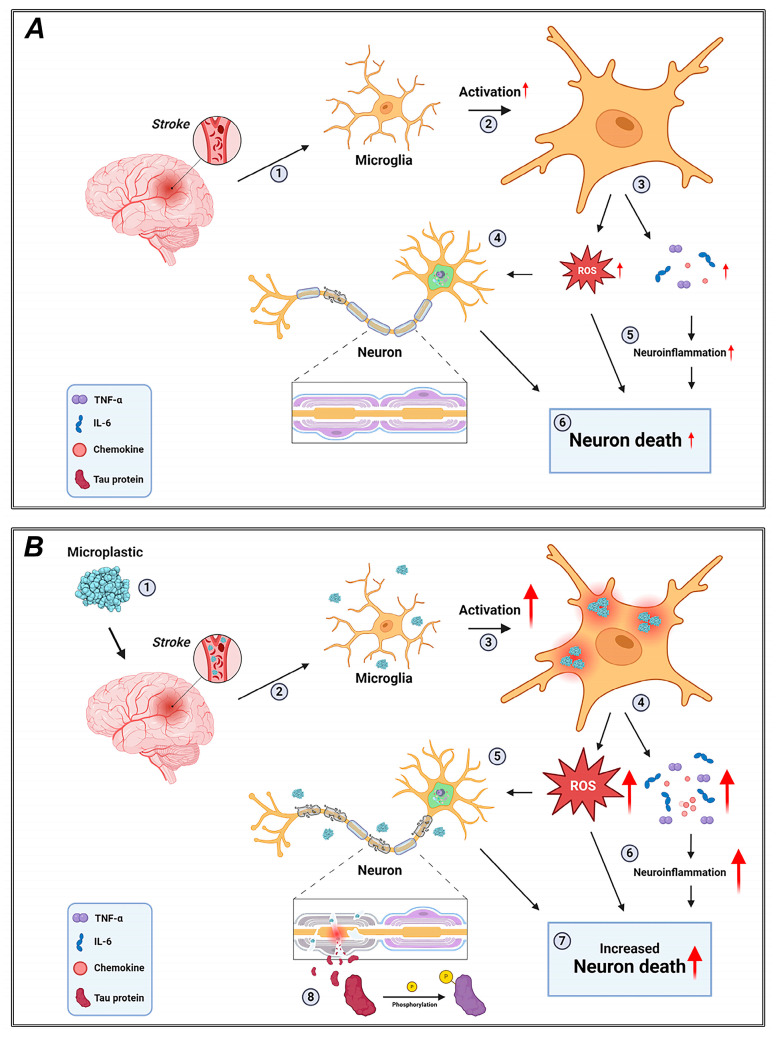
Proposed mechanism of neuronal damage caused by microplastics in GCI. (**A**) Standard ischemia model. (**B**) Ischemia model in conjunction with microplastic. Microplastics smaller than 2 μm can enter the human body [44], travel through the bloodstream, and directly penetrate the brain via blood vessels and lymphatic vessels, penetrating the blood–brain Barrier (BBB). Subsequently, they cause damage to the vessel walls. They accumulate within cells through phagocytic actions of microglia, leading to excessive activation and increased neuroinflammation by over-releasing cytokines and chemokines, such as those in the Interleukin family. Additionally, they approach neurons and induce cognitive impairment and cell death by damaging myelin sheaths and reducing intercellular signal transmission, including electrical signals. Furthermore, they cause damage to microtubules, releasing various proteins, including tau, outside the cells. Tau proteins can undergo phosphorylation due to various factors, and this continuous cycle may lead to the development of Alzheimer’s disease. In conclusion, microplastics directly infiltrate the brain, inducing increased neuroinflammation, cognitive impairment, and neuronal death through mechanisms such as damaging myelin sheaths and microtubules. “Created with BioRender.com”.

Cerebral ischemia, characterized by insufficient blood flow to the brain, is becoming increasingly prevalent in aging societies and is often associated with a variety of factors, including age-related vascular changes, lifestyle choices, and environmental exposures [45]. The potential health impacts of microplastics, and in particular their potential contribution to cerebrovascular disease, is another area of emerging interest.

Microplastics, defined as plastic particles smaller than 5 mm, are widespread in the environment due to widespread plastic use and improper waste management. These particles can enter the human body through inhalation, ingestion of contaminated food and water, and skin contact. Once in the body, microplastics can cause oxidative stress, inflammation, and other cellular damage, leading to a variety of health problems [46,47]. Recent studies have begun to identify potential links between microplastic exposure and various neurological and vascular diseases, including ischemia, tauopathy, and myelin damage [48]. In this study, we explore the potential mechanisms through which microplastics can affect these diseases (Figure 5).

Polystyrene beads (0.5 µm in size) were used to investigate the effects of orally administered microplastics on ischemic damage. However, the use of polystyrene beads in this research has limitations in fully representing the diverse sizes, shapes, and chemical compositions of microplastics found in the environment. In real-world scenarios, microplastics range in size from nanometers to millimeters and can take various forms, such as fibers and fragments, potentially leading to varying biological responses.

Despite these limitations, the study provides significant insights into the potential impacts of microplastic exposure on human health. First, the findings reinforce the link between environmental factors and human health, suggesting that microplastic toxicity is not merely an issue of accumulation but may also exacerbate acute conditions, such as ischemic damage. Second, microplastics were found to increase the secretion of inflammatory cytokines (e.g., IL-6, TNF-α), which could impair tissue recovery and exacerbate damage at ischemic sites. Lastly, for patients with ischemic diseases, microplastic exposure may worsen outcomes, such as cognitive decline, highlighting the need to develop therapeutic strategies that address such environmental exposures.

The effects of microplastics on ischemic cell death were specifically analyzed using polystyrene beads. Previous studies have indicated that external, non-digestible particles can negatively impact ischemic cell death. This study contributes by examining the role of microplastics (0.5 µm polystyrene beads) in a global ischemia animal model. However, the findings do not rule out the possibility that other particulate matter may exert similar physiological effects. For example, fine particulate matter (PM2.5) and other environmental particulates are known to cause inflammation and tissue damage. While this study highlights the unique biological effects of microplastics, it also underscores the need for comparative research involving other types of particulate matter (Figure 1).

Cerebral ischemia can be categorized into primary neurological damage caused by blocked blood vessels and secondary neurological damage caused by reperfusion. This process damages microtubules, induces an inflammatory response, and ultimately leads to neuronal cell death [49]. Excessive exposure to microplastics can exacerbate this damage. The main mechanism involves the activation of microglia, which is over-activated by microplastics [50]. Microglia activated by cerebral ischemia recognize excessive microplastics as a pathogenic factor and become more activated, resulting in a severe inflammatory response. This activation releases numerous pro-inflammatory mediators and chemokines, including IL-6 and TNF-α [51], which play a role in neuronal death associated with cerebral ischemia and worsen the condition [52].

The results showed that continuous ingestion of microplastics at a concentration of 50 mg/kg for a week before inducing cerebral ischemia resulted in increased neuronal cell death and increased microglial activity. Western blot analysis confirmed elevated levels of the pro-inflammatory cytokines IL-6 and TNF-α. These results suggest that inflammatory cytokines and chemokines such as IL-6 and TNF-α are important in regulating tau phosphorylation patterns during the early stages of tau degeneration. In addition, these cytokines can affect the function and structure of tau protein, indicating that microglia-specific neuroinflammation may promote tau pathology and lead to neurodegeneration (Figure 2).

Neurons are highly dependent on microtubules for the transportation of organelles and synaptic vesicles [53]. Secondary damage caused by reperfusion after cerebral ischemia leads to a significant loss of microtubules, resulting in cognitive and memory impairment [54]. Intervention of microplastics exacerbates this loss. Previous studies have shown that myelin sheath, a component associated with microtubules, is significantly damaged by physical abrasion caused by microplastics [35], leading to impaired signal transduction. Based on these findings, we sought to determine the extent of microtubule loss and the degradation of the lipid components that comprise microtubules following excessive microplastic ingestion and induced cerebral ischemia.

Furthermore, we also sought to determine the extent to which tau protein, which maintains the stability of microtubules and myelin sheaths, is phosphorylated and cleaved due to physical damage caused by microplastics and reperfusion of cerebral ischemia. The ultimate goal was to investigate the formation of neurofibrillary tangles and assess cognitive function. The results showed that inducing cerebral ischemia after a week of excessive microplastic ingestion significantly reduced microtubule-associated protein 2 (MAP2) and myelin basic protein (MBP), as confirmed by immunohistochemistry. Western blot analysis also confirmed a significant increase in phosphorylation and cleavage of tau protein under these conditions (Figure 3).

Behavioral assessments using the modified neurological severity score (mNSS) and Morris water maze (MWM) test further corroborated the findings: inducing cerebral ischemia after excessive microplastic ingestion significantly impaired both motor and cognitive performance, highlighting the close relationship between microplastic exposure and the worsening of Alzheimer’s disease and other neurological disorder-like conditions (Figure 4).

Contrary to expectations, many observed outcomes were primarily driven by global cerebral ischemia (GCI) itself, as the differences between the GCI-Veh and GCI-MP groups were relatively subtle. This suggests that while microplastics may exacerbate ischemic injury, they are unlikely to be primary drivers. Additionally, the potential impact of microplastics on tau phosphorylation might have been overshadowed by the dominant effects of global ischemia under the experimental conditions. The exposure level and duration of microplastics in this study may not have been sufficient to produce more pronounced effects on tau phosphorylation.

A high dose of 50 mg/kg, equivalent to 3.5 g for a standard 70 kg adult male, was used in the experiment. Although data on microplastic accumulation in humans remain limited, typical exposure through food and beverages is significantly lower than the dose used in this study. This underscores the need for future research exploring the effects of lower, more physiologically relevant doses to bridge the gap between experimental models and real-world human exposure.

Further investigation is needed to understand how factors such as microplastic dose and size contribute to physiological responses in ischemic injury models. Exploring dose- and time-dependent effects of microplastics on tau phosphorylation and their interactions with ischemic damage will be critical for elucidating the complex impacts of particulate matter on ischemic injury.

This study is the first to systematically demonstrate that microplastic exposure, via forced ingestion of polystyrene beads in a rodent model, can induce significant physiological changes in vivo. It provides fundamental insights into the biological effects of microplastics and highlights previously unaddressed potential risks. By doing so, it offers crucial scientific evidence for assessing the health impacts of microplastics and shaping related policy recommendations.

## 5. Conclusions

In conclusion, the potential for microplastics to damage microtubules and cause disease is a significant concern for public health. The mechanisms through which microplastics destabilize microtubules include physical abrasion and interference with tubulin polymerization, which contribute to a variety of diseases, particularly neurodegenerative disorders. More research is needed to fully characterize the pathways through which microplastics exert their toxic effects and to develop interventions to protect human health. This includes understanding how microplastics interact with cellular structures, identifying biomarkers for early detection of microplastic-related damage, and developing strategies to mitigate exposure and impacts. Public awareness and policy action are also essential to reduce plastic pollution and its health effects.

## Data Availability

All data generated and analyses performed during this study are included in this published article.
